# Barriers and Facilitators to the 3 Sides of Extended Reality-Rehabilitation Adoption: Scoping Review

**DOI:** 10.2196/80055

**Published:** 2026-05-20

**Authors:** Luuk Baltissen, Miranda Stienstra, Lien Denoo, Joris Knoben

**Affiliations:** 1 Department Strategy and Entrepreneurship, Tilburg School of Economics and Management, Tilburg University, Warandelaan 2, Tilburg, 5037 AB, The Netherlands, 31 134664720

**Keywords:** scoping review, extended reality, virtual reality, adoption behavior, rehabilitation care

## Abstract

**Background:**

Rehabilitation using extended reality (XR) technologies can be used to address the growing shortage of health care staff, but the adoption level remains low. The current literature has given some first insights into what drives patients and clinicians to (not yet) adopt XR-rehabilitation tools. However, it does not sufficiently take into account that these tools will only be widely adopted if 3 types of actors collectively commit to it: developers must develop a tool, clinicians must prescribe it, and patients must use it. Because the preferences of these 3 actors may not always align, we aim to provide the first multi-actor insight into adoption.

**Objective:**

This research aims (1) to determine what drives patients, clinicians, and developers to adopt or develop XR-rehabilitation tools and (2) to determine if and how these drivers align or misalign.

**Methods:**

We searched PubMed, Embase, SCOPUS, and Web of Science with four search term categories: (1) types of rehabilitation care, (2) XR technologies, (3) adoption constructs, and (4) behavioral drivers. Using these search terms, we identified 1164 results, of which we included 64 in our review. All relevant empirical results within these papers were structured using the Non-adoption, Abandonment, Scale-up, Spread, and Sustainability (NASSS) framework.

**Results:**

After exploring the adoption drivers of patients, clinicians, and developers, we identified 3 potential misalignments among these actors. The first possible misalignment is that clinicians may have much higher standards for a tool’s medical efficacy. Because of this, they refuse to prescribe a medically less effective tool that would have matched the experience needs of patients and developers. The second possible misalignment is that clinicians value their work experience, while this is not a relevant factor for patients. Because using XR-rehabilitation tools can negatively impact a clinician’s work experience, they may decide not to use a tool that patients and developers would have liked to use and develop. The third possible misalignment is that the patients’ and clinicians’ limited ability or willingness to pay may hinder the developer’s economies of scale. Developers currently face high development costs, which they can recover by letting patients or clinicians pay for the tool. But these actors are not always able or willing to do so. As a result, developers may struggle to gain profitability, which limits the supply of XR tools.

**Conclusions:**

Our scoping review provides initial evidence that differences in the behavioral drivers of patients, clinicians, and developers may lead to misalignments that hinder the adoption of XR-based rehabilitation tools. Scholars can use this review to further investigate potential misalignments between relevant stakeholders and how to resolve them. We encourage developers and regulatory institutes to collaboratively investigate the feasibility of new revenue models and product offerings to increase the adoption of XR-rehabilitation tools.

## Introduction

By 2035, a global shortage of 12.9 million health care workers is predicted [1]. Such a shortage will have tremendous negative societal consequences, such as decreased quality of care and increased staff burnout [2,3]. Rehabilitation care is particularly vulnerable to this problem, as demand in this sector is exceptionally high. A total of 1 in 3 people will need it at least once in their lifetime, and the therapy is often long and labor-intensive [4,5]. Because the workforce cannot be increased sufficiently, the sector must also look at technological innovations to reduce the workload. For instance, the Dutch Minister of Public Health stated:*“*With smart innovations, we can provide the care and support that is needed, thereby relieving the burden on informal caregivers and employees*”* [6].

One of these smart innovations is using extended reality (XR) as a tool for in-person and telerehabilitation. XR is an umbrella term encompassing virtual, augmented, and mixed reality (VR, AR, and MR) technologies that create user experiences where “real-world” elements are enhanced or replaced by “virtual” elements [7,8]. Telerehabilitation entails the delivery of rehabilitation services at a distance. Using these technologies can enhance the patient’s therapy enjoyment and adherence while reducing the treatment duration [9,10]. However, despite these potential benefits and initial proof of the therapy’s medical effectiveness [9], less than 5% of clinicians use it in their clinical practice [11-13]. Due to this, the potential of XR in rehabilitation care is far from realized.

Various papers have recently illustrated what drives patients and clinicians to not yet adopt XR-rehabilitation tools [14-16]. For example, the tools are deemed too expensive and often contain technical defects. Additionally, some patients need too much time to get used to the tool, and some clinicians believe that the movements patients make in XR differ too much from real-world movements.

Although these papers have provided a valuable first insight into nonadoption, they do not fully highlight the interconnectedness of both actors’ adoption decisions. As one paper [16] recently stated: “Since the majority of studies have a singular perspective, there is a need to redirect the research focus towards a more comprehensive approach within the patient’s environment.”

Because clinicians are gatekeepers to the patient’s access to care, adoption of the XR tool is inherently a collective decision by *both* actors [17,18]. To make it even more complex, the demand of these 2 actors can only be fulfilled if there are people willing to develop and sell the required XR tools (hereafter called developers).

Due to this need for supply, XR-rehabilitation tools will only be widely adopted if all 3 actors commit to it: developers must develop a tool, clinicians must prescribe it, and patients must use it. But their preferences and incentives do not always align with each other. For example, the clinician’s informational advantage over their patients means that their (non)adoption decision can partially be driven by factors that patients do not consider or by clinician self-interest [19,20]. Developers are also in a buyer-supplier relationship with both patients and clinicians [21].

Because such misalignments can cause one actor to not opt in, they can prevent adoption for all. It is therefore essential to understand the drivers of all 3 actors as well as how they align and misalign. Yet, the current unilateral view on adoption does not allow for this [22]. Thus, to get the first multi-actor insight into adoption, we conducted a scoping review with the following objectives [23]:

1. Determine what drives patients, clinicians, and developers to adopt or develop XR-rehabilitation tools.
2. Determine if and how these drivers align or misalign

## Methods

This work was conducted according to the Preferred Reporting Items for Systematic reviews and Meta-Analyses extension for Scoping Reviews (PRISMA-ScR) reporting guidelines [24]. The filled-in PRISMA-ScR checklist can be found in Checklist 1. To ensure adequate and efficient database coverage for published academic papers, we searched Scopus, Embase, Web of Science, and PubMed from October 31, 2024, to January 20, 2026 [25].

We used 4 search term categories in these databases. The first category covers the main types of rehabilitation care. As Battel et al and others recently illustrated, rehabilitation is a very broad field that largely overlaps with other medical specialties like psychiatry, geriatrics, and palliative care [26-29]. To cover the broad range of relevant studies, we searched for the general term “rehab*” and included papers from all overlapping subfields, such as psychiatric and neurological rehabilitation. We also searched for the 4 therapies most commonly classified under (XR-)rehabilitation: occupational, physical, speech, and language therapy [14,16,30]. However, to avoid capturing studies that solely focus on the other specialties, we did *not* search for terms like psychiatry and neurology. The second category covers XR types. Currently, the literature views (too) many gaming devices as an XR tool, which has made it difficult to compare different studies [31-33]. To enhance comparability, we follow Milgram and Kishino’s view on XR and only consider a device an XR tool if it generates a user experience where “real-world” elements are enhanced or replaced by “virtual” elements [7,8]. We therefore searched for the primary types of XR, head-mounted devices (HMDs), and exergames, but *not* for devices like the Wii, Xbox Kinect, and Virtual Reality Rehabilitation System. The third category comprises terms that are associated with adoption [34]. In this review, we define adoption as “the intention, initial decision, or action to try or employ an innovation or evidence-based practice” [34]. This term is often interchanged with similar constructs like uptake and acceptance, which are salient to all stakeholders (including developers) [34]. To capture this breadth, we included 42 different adoption-related terms in our search string [34]. Importantly, several of these terms, such as “feasibility,” “integration,” and “adherence,” are also often used to refer to a tool’s medical efficacy and technical operability [35-38]. Because these topics fall outside the scope of this review, we aimed to remove them a priori by using a fourth search term category that captures behavioral drivers [39,40]. As Nilsen [41] illustrated, the determinant framework is the only theoretical approach that investigates adoption behavior by explaining how different variables influence (ie, drive) adoption. Because this aligns with our research objective, we added all deterministic terms that Nilsen identified [41]. This includes terms such as barrier* and facilitator*. We did *not* search for terms such as obstacle* and challenge*, because they are also frequently used in other contexts. For example, patients often face health challenges due to their disorder. The full search query can be found in Multimedia Appendix 1. Multimedia Appendix 1 also outlines how the query was progressively extended from the first to the last search date in response to reviewer and editor feedback.

Using the criteria in Textbox 1, the first author (LB) selected all resulting papers in three rounds: (1) title, (2) abstract, and (3) full text. To illustrate the reliability of the screening process, the second, third, and fourth authors (MS, LD, and JK) all blindly screened a unique and randomly selected set of at least 10% of the papers *each*. This resulted in a coverage of over 30%, which goes well beyond the typical 10%‐25% requirements for a trustworthy estimate of the interrater reliability (IRR) [42]. The first author held individual meetings with all authors after they completed each stage. In these meetings, they discussed and resolved any discrepancies that emerged. The outcomes of these meetings were directly communicated to all other authors. We measured IRR using Cohen’s Kappa [43,44].

Textbox 1. **All inclusion and exclusion criteria that were applied during the screening process.**

**Inclusion criteria**

- Available in full text
- Written in English
- Peer-reviewed papers
- Research in the rehabilitation context, both in-clinic and at-home, including all subfields that overlap with other medical specialties (eg, neurological or psychological rehabilitation)
- Research on a medical application of extended reality
- Empirical data on the behavioral drivers of developers, clinicians, or patients

**Exclusion criteria**

- No full text available
- Not written in English
- Works that are not peer-reviewed papers (eg, conference proceedings)
- Research in a nonrehabilitation context (eg, distracting patients before surgery or providing visual enhancement)
- Research on different technologies (artificial intelligence, robotics, etc) or non–extended reality devices (Wii, Xbox Kinect, etc)
- No empirical data (eg, literature review) or data with no insight into the individual behavioral drivers (eg, only including a System Usability Score or aggregating data across actors)

A data charting form was developed by all researchers to map the descriptive variables reported in the selected papers. This entailed the actors that were treated, the type of empirical data, the adoption framework used, the usage location, the intervention type, the disorders treated, the XR-type, the device used, and the funding source.

To structure the empirical findings of the selected papers, we divided all data across 3 groups, based on the actors they apply to (ie, patients, clinicians, and developers). After this, the data for each actor group was divided into 7 subgroups based on the domains in the Non-adoption, Abandonment, Scale-up, Spread, and Sustainability (NASSS) framework (ie, 21 subdomains in total). This framework explains the Nonadoption, Abandonment and challenges to Scale-up, Spread, and Sustainability of innovations using the interaction between (1) the patient’s condition, (2) the technology, (3) the value proposition, (4) the adopter system, (5) the health care organization, (6) the wider system, and (7) how these 6 domains develop over time [45,46]. We chose this specific framework because it is the adoption-related framework that best captures the complex interaction between patients, clinicians, and developers [41,46-48]. To get a more granular insight into these NASSS domains, we then used the guidelines of Colquhoun et al [49] to inductively draft a set of categories that best represented the dynamics within each domain. The first author (LB) used this structuring form to determine whether the categories were present (1) or absent (0) in each paper. To ensure reliability of this process, the second, third, and fourth authors (MS, LD, and JK) reviewed the first author’s (LB) completed form for all papers they included in their screening process. They also assigned a “1” to a category if it was present in a paper, and a “0” if it was not. Here, the first author (LB) also held individual meetings to discuss any discrepancies. Because the data structuring form contains a large number of categories, there were many zeros in some papers. Because Cohen Kappa can yield deceptively low values in such cases of class imbalance, we used Gwet AC1 as the IRR metric instead [50,51]. To further substantiate our findings, we also conducted a thematic frequency count to illustrate whether and how the prevalence of themes differed between actors [52,53].

## Results

### The Literature Selection and Structuring Process

**Figure 1.**
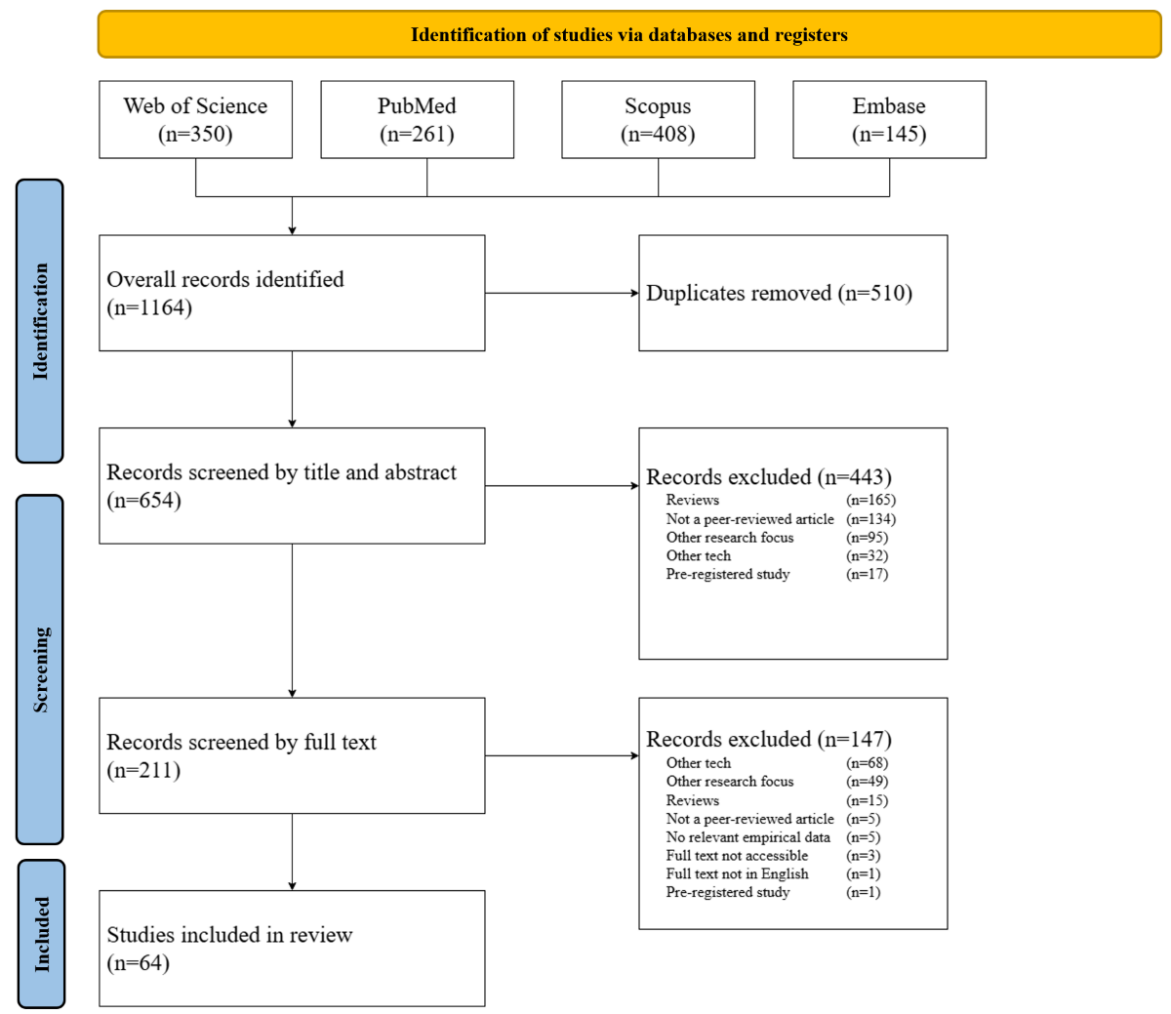
Study selection flow diagram.

The search query yielded 654 unique records, of which 64 have been included in this review to analyze the behavioral drivers of patients, clinicians, and developers. The main exclusion reasons were that the work was a literature review (n=180) or that it had a different research focus, such as clinical trials or technical proofs-of-concept (n=144). Other reasons for exclusion were that the work was not a peer-reviewed article (n=139) or that it focused on non-XR devices (n=100). A total of 27 records were removed for different reasons, mostly because they were preregistered but not yet executed (n=18). Figure 1 outlines the full selection process. MS, LD, and JK validated LB’s screening and charting process for 219 of the 654 (33.5%) initially screened records. This validation process indicated that LB selected the literature nearly perfectly in both the title and abstract stage (κ=0.83) and the full paper stage (κ=0.89) [54]. The empirical findings were also charted nearly perfectly, with the average Gwet AC1 being 0.87 (range 0.63-1.00). Most studies were funded by academic, health care, and/or governmental institutions (48/64, 75%). In total, out of 64 studies, 11 (17.2%) studies did not receive external or research-specific funding, while 5 (7.8%) studies did not disclose their funding source in the paper. No studies were directly funded by firms from the life sciences industry, although some funds were provided by industry-adjacent sources like EUROSTARS. Further details can be found in Multimedia Appendix 2.

**Figure 2.**
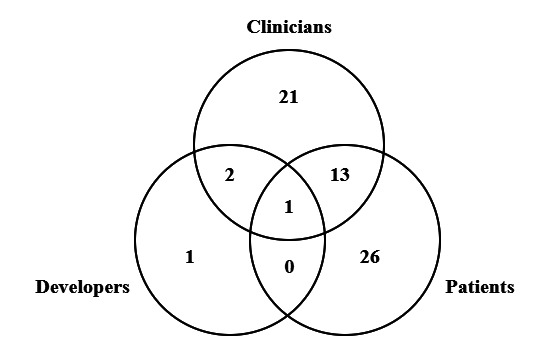
The number of papers included per actor.

As shown in Figure 2, the developer’s perspective is highly underexposed in the included papers. Patients and clinicians were treated in 62.5% (40/64) and 57.8% (37/64) of the papers, respectively, while developers were mentioned in only 6.3% (4/64). Additionally, only 25% (16/64) took a multi-actor approach, and Saidi et al [22] is the only paper that included all 3 actors. In their study, Saidi et al conducted 3 workshops and 10 interviews with patients, clinicians, and developers in stroke rehabilitation. Importantly, however, they aggregated most findings across actors and mostly referred to all 3 groups as “participants.” As a result, their analysis offered limited insight into how the drivers differed across actor types. This further supports the need for a 3-sided perspective on adopting XR-rehabilitation tools. The descriptives of each included paper can be found in Multimedia Appendix 2.

### Domain 1: The Value Proposition

#### Overview

This domain addresses whether a new technology is worth developing in the first place, and for whom it generates value [45]. We interpret this domain as the net sum of benefits and losses. XR-rehabilitation is more likely to be adopted if the benefits of developing or adopting the tools outweigh the associated losses for *all* actors. Conversely, if at least 1 actor faces insufficient benefits, then that actor will not opt in and may prevent adoption for all. We outline the benefits and losses for all 3 actors individually.

#### Patients

Overall, the benefits and losses for patients can be divided into three categories: (1) care experience, (2) medical efficacy, and (3) savings or investments of time and money.

##### Care Experience

The most frequently mentioned category is the care experience (31/40 papers, 77.5%). Patients see numerous benefits of using XR tools. First, they make therapy more fun [31,55-83]. Currently, clinicians ask patients to do a certain set of exercises, which they must individually repeat at home. The repetitiveness makes these exercises dull, causing low adherence. At-home XR provides a way to alleviate this dullness. In XR, exercises are presented as a game that patients can play by themselves or against others. These exergames are interactive and provide feedback on their performance, which makes exercising more fun. Second, XR tools can give patients more autonomy [55,58,62,64,69,72,75,84]. Currently, patients must frequently meet their clinician for exercise instructions or supervised practice. These visits can interfere with the patient’s daily activities. At-home XR tools can address this issue by providing exercise demonstrations and enabling patients to exercise at a convenient time and location. This allows the exercise schedule to better fit into the patient’s daily life. Some additional gains are that the tool’s biometric feedback can give patients insights into what they are capable of, can be relaxing, can give them confidence, can make them feel that they are more in touch with their caregivers, and allow them to work with novel tools [55,58,59,61,62,64,66,70,73,83,84].

Unlike the benefits, relatively few patient papers mention losses related to the care experience [55-57,62,64,70,72,74,76,80]. Only two losses were mentioned more than once: (1) frustration due to the inability to exercise effectively or to win, and (2) a feeling that therapy should be challenging rather than fun. We outline both losses below.

Frustration generally arises from poorly designed hardware and software. For example, bugs and glitches take patients out of the immersive flow and require time to resolve [57,61,72]. A poor design can also lower the patient’s self-image [56,64,74,80]. Because of their impairment, some patients may find the exergames too difficult to play, may be unable to use the hardware, or may continually lose against “able-bodied” friends and family. This can make them feel limited in what they can do and possibly inferior to others. One patient excellently illustrated this point by stating that *“dealing with the frustration of using VR combined with the existing frustration of having aphasia and trying to communicate”* makes them *“feel like a total failure”* [74].

Additionally, 2 papers indicated that patients felt that the enjoyable nature of VR was inconsistent with their expectations of physiotherapy [70,76]. One patient indicated that *“*you have an expectation that when you go to [physical therapy] they give you exercises or manual treatment so when you leave it feels like you’ve done something*”* [76].

By replacing these regular exercises with enjoyable experiences, some patients feel they no longer receive any treatment.

##### Medical Efficacy

A total of 67.5% (27/40) of the patient papers illustrated that medical benefits are a major adoption driver. However, unlike the care experience, there is not one benefit that is very frequently mentioned in this category. Rather, 14 [22,55-58,60,64,66,69,70,76,80,82,85] papers mention that XR-rehabilitation tools allow patients to move more due to the motivational aspect and more opportunities to train by themselves. Additionally, 12 [55,57,58,68-70,73,76,81-84] papers mentioned that XR tools can distract patients from their pain. This is a medical benefit in itself, but it also leads to a lower fear of movement and thus better exercise execution. Other benefits were that patients can practice in a more controlled environment than the real world [59,61,74,79,86] and that they can better visualize their exercises [61,69,72,80]. Using XR tools can also reduce anxiety or stress symptoms [59,62,65], offer clinicians more (objective) insights into the patient’s performance through tracking [55,72], stimulate patients cognitively [80,83], and allow them to exercise independently when they are still on the waiting list [55].

Although XR-rehabilitation is designed to provide medical aid to patients, it can also have substantial medical downsides. When using XR technology, some patients can feel nauseous, claustrophobic, or disoriented [31,55-59,61-63,66,70,71,73,74,79-83,87,88]. Others feel unsafe exercising using immersive HMDs because they cannot see their surroundings [22,58,70,71,80,89], feel that they may make wrong movements due to overexcitement or lack of supervision [55,64,80], or are afraid of becoming addicted to the exergame [59].

##### Savings or Investments of Time and Money

The last value proposition for patients is the savings or investments of time and money. On the one hand, XR tools can save patients time because they can rehabilitate at home, rather than having to travel to their clinician [55,57,64,74]. Conversely, using XR-rehabilitation tools often requires an upfront investment [55,57,59,61,62,64,69-74,77,78,80,83,84,88-90]. To be able to use the tool, patients must often spend time getting to understand it, setting it up, and fixing bugs when needed. They must also sometimes pay an additional fee for the hardware and software.

### Clinicians

While assessing the value propositions for clinicians, it became apparent that they largely overlap with those of patients, because both actors value (1) the tool’s medical efficacy, (2) the patient’s care experience, and (3) investments or savings in time and money. However, despite these similarities, 2 differences appeared.

The first difference is that patients and clinicians evaluate the 3 value propositions differently. When evaluating the tool’s medical efficacy, clinicians consider a broader set of factors than patients. Particularly, they are more likely to also consider the long-term risks of overcompensation, compensatory movements, infections, attachment issues, and the lower quality of tele-evaluations [10,11,13,56,66,91-101]. When evaluating the cost (savings), clinicians go beyond considering the patient’s costs by also considering the costs they must bear themselves. These clinician costs include both time (eg, learning to use the tool) and money (eg, maintenance costs) [10-13,31,56,58,68,73,79,81,90-92,94-96,98-100,102-105]. When evaluating the patient’s care experience, clinicians appear to value fun differently than patients do. As noted earlier, XR can be fun for patients because they do not have to do “dull” exercises, can play with friends or family, and get performance feedback. Clinicians acknowledge these aspects [10,13,56,58,81,82,92,101]. However, they seemingly perceive fun as a means to enhance therapeutic engagement and clinical outcomes, rather than a valuable end in itself. For example, one physiotherapist stated that *“for compliance – if it’s fun compliance is going to go up”* [95]. Another therapist *“felt that VR helps to improve the child’s engagement, which can facilitate movements, increase movement control and facilitate stability and function.”* [56] One patient, on the other hand, stated that “*If [occupational or physical therapy] was pretty boring then a lot of the times I would just be like ‘okay, well, let me just get through this then I’ll just be able to do VR.”* [68]. This suggests that while both groups recognize the benefits of enjoyable experiences, patients can also value them for their own sake. It is important to note that this is not a strict dichotomy; clinicians still appreciate the experiential benefits of XR. However, the current literature does indicate that they value it less than patients do.

The second difference is that patients do not refer to the clinician’s work experience. Although clinicians also rarely mention it themselves, XR tools can make their work more enjoyable because they can work independently of time and location, feel innovative, and can offer a more varied set of treatments [10,11,13,31,84,91,92,96,97,103,105]. However, XR tools can also make the clinician’s work less enjoyable, as using them can make them feel like a virtual life coach or a patient mill, can narrow their skillset, or can awkwardly interrupt the in-clinic session [13,91,97,100,103].

### Developers

Of all 64 included papers, only 4 treated the developer’s perspective [22,94,96,106]. Three of these papers did not truly focus on their development barriers or facilitators, but instead used their input to validate the feasibility and relevance of the clinicians’ ideas on XR usage [22,94,96]. For example, in 1 paper, VR specialists “engaged in discussions with the [clinicians] about how VR tasks could be programmed to incorporate more or less challenging situations and distractions” [96]. Only the paper of Kulkov et al [106] had the developer’s perspective at its core. Because of the limited number of developer-focused papers, we only found 2 indications about what the benefits and losses for developers could be.

First, 3 [94,96,106] papers indicated that developers seem to reasonably understand the benefits and losses for patients and clinicians. For example, developers mentioned that XR “provide an indoor solution to motivate physical activity, which could be achieved on a ‘daily basis’” [94], “is good for building an environment that duplicates the environment people are going to be in” [96] and that “self-treatment could be offered at a convenient time and place with follow-up visits to the specialist” [106]. In 2 [96,106] papers, developers also acknowledged that XR requires high financial investments from patients and clinicians and that clinicians may feel less needed or relevant when using XR. Although developers did not mention all value propositions emphasized by patients and clinicians, they also did not mention any new or different ones. This suggests that developers have a fair understanding of the user’s preferences.

The second main insight is that developers face *“costs of development [that] are still pretty high at present”* [94]. Because developers can only sustainably supply tools if they at least break even, it is plausible that these costs are a substantial barrier for them.

### Domain 2: The Condition

This domain covers the patient’s condition, sociocultural factors, and comorbidities. Because the developer papers did not dive into this, their perspective is not outlined below.

#### Patients

Of the 19 [56-62,64,66,69,71,72,74,75,79,81,87,88,90] patient papers that touch upon the condition domain, there is one major consensus: more impaired patients are less likely to adopt XR-rehabilitation. These patients generally have less confidence in their ability to use the tools. They are also less able to use them, even if they wanted to. As one stroke patient stated: *“*I don’t think I would do anything because... like my balance still isn’t wonderful*”* [71].

There are also indications that patients who are “too healthy” are less likely to use XR tools, because they benefit more from exercising in the real world [58,64,74]. However, because this point is sparsely mentioned, there is less widespread consensus on this aspect. Additionally, although 6 [31,60,61,66,87,90] papers included multiple disorder types, there is no definitive indication of whether patients with certain disorder types are more likely to adopt XR tools than others.

#### Clinicians

A total of 48.6% (18/37) of the clinician papers touch upon this domain. Twelve of these [56,58,66,81,87,92,94,96,98,99,102,103] also emphasize that clinicians will not prescribe XR tools to patients who are “too impaired,” because they cannot safely use them. Additionally, while 10 [11-13,97,103,105,107-110] papers included multiple disorder types, there is again no definitive indication of which disorders clinicians are more likely to target with XR tools.

One aspect highlighted in the clinician papers, but not in the patient papers, is that clinicians require a minimum number of patients using an XR tool before they are willing to adopt it [11,92,96,99,100,103,105,107-109]. As one clinician said: “[Due to h]igh acquisition costs, the patient group for which [using XR] makes sense is too small to justify a purchase for me” [11].

Because using XR requires high financial and time investments, the tool must be used by enough patients to provide value. Consequently, clinicians who work with high-prevalence conditions are more likely to adopt XR tools, given the larger potential user base.

### Domain 3: The Technology

This domain treats all features, knowledge generated or needed, and supply chain issues of the XR-rehabilitation technologies. Although the included papers treated several technologies, the HMD was the most used [10,11,22,31,55,57-65,67-70,73-83,85-88,90,94-96,98-101,111,112].

#### Patients

A total of 90% (36/40) of the patient papers mentioned technological factors that influence the patient’s adoption decision. There is a broad consensus on the importance of 6 such factors. The most mentioned feature is content variety and customization [22,31,55-67,71,74,77,79-82,84,86,88,111]. Patients want to use tools that fit their (changing) needs, and they want to enjoy a wide range of games to stay entertained. The second-most mentioned feature was the presence of a support staff [55,57,59,60,62-66,68,69,71,73,74,76-80,84,87]. Even when exercising at home, patients want to stay in touch with clinicians for medical advice and supervision. They also want to be able to contact someone for technical support. Comfort and ease of use were the third and fourth-most mentioned features [31,55-59,61,62,66,69,72-74,76-80,82,84-88]. Devices that are uncomfortable to wear or difficult to use can cause physical strain, enhance the patient’s sense of impairment, and possibly prevent patients from continuing their therapy. The 2 other frequently mentioned features are technological reliability and the availability of tutorials or guidelines [31,55-57,59-62,64-67,71,72,74,79-81,84,87,88]. Tools that are buggy are less enjoyable and stimulate patients to stop using the tool. Additionally, because most patients are new to XR, they want to be educated on how to use it.

There were also 2 features on which the findings were inconsistent. The first one is the level of immersion. Some patients prefer using an HMD with immersive VR because they are more engaging [31,55,57,68,73,74,79,81]. Others prefer nonimmersive HMDs because their inability to see the outside world makes them claustrophobic or afraid of hitting something in their physical surroundings [57,58,70,73,74,79]. It is unclear whether the benefits of immersion outweigh the risks and how these potentially differ between patients. The second inconsistent feature is the exercise type. Some patients find full-body movements with free movement more engaging than “simple” movement repetitions [68,71,74,76,89]. However, these full-body movements come at a higher risk of falling or tripping over objects. Therefore, some patients prefer seated exercises with “simple” movement repetitions [57,71,74,89]. In total, 6 papers illustrate the patient’s preference for at least 1 of the 2 exercise types [57,68,71,74,76,89], with 3 of those mentioning both [71,74,89]. However, none of the works truly indicate which exercises patients prefer, or whether preferences differ between patients.

#### Clinicians

A total of 97.3% (36/37) of the clinician papers mentioned XR features that influence whether clinicians adopt the XR tool. When comparing these to the patient’s technology factors, it became apparent that both patients and clinicians value training or guidelines [10,11,13,56,58,66,68,81,82,84,85,87,91-93,95,96,98,99,101-105,107-110], software variety or customization [10,11,13,22,56,66,68,73,79,81,82,85,91,92,94,96,98,99,101-103,107-110], ease of use [11,12,31,56,66,73,81,82,84,85,91,93,96,97,99,101-103,105,107-109], support staff [11,13,22,31,56,68,73,82,87,92,96,98,99,102,103,105,107-109], tool reliability [10,11,31,56,66,81,82,84,92,94,97,99,101,104], and comfort [11,56,66,68,82,91,94,95,98,99,101]. There is, however, one main difference between patients and clinicians.

Namely, clinicians have a much stronger preference for technologies with low (medical) risks. Three aspects stand out in particular. First, clinicians mention guidelines and medical evidence much more than patients. Guidelines were mentioned 28 times by clinicians versus 13 times by patients. Medical evidence was mentioned 18 times by clinicians [10,11,13,56,58,91,92,96-100,103,105,107-110], and only once by patients [74]. Second, clinicians expect more depth in their guidelines and evidence. Of the 13 papers that treat both patients and clinicians [31,56,58,66,68,73,79,81,82,84,85,87,90], 4 explicitly referred to their guideline preferences [56,66,84,87]. Patients are generally satisfied with an explanation of the tool’s potential benefits and practical tutorials on how to use it. For example, one patient stated that they want *“a field training module [with] the basics of how to connect to the Internet, Bluetooth, troubleshooting”* [56]. However, clinicians want more than this “everyday” information, because they see more risks and feel responsible for the patient’s well-being. Hence, they also want information on treatment dosages, side effects, etc. For example, one work [56] illustrated that clinicians *“would like just some guidance on what do with [the patient]and how far to push them”* and that they *“would want to always ensure that [they]’ve demonstrated and that [the patient] is using it in the correct way”*. The third indication of the clinician’s risk-aversion is that all clinician papers prefer seated exercises because they are safer than full-body exercises [22,94-96,99]. Some patients, however, have the exact opposite preference, because full-body movements are more fun to do.

#### Developers

Only 1 [96] paper gave an insight into the developer’s perspective on XR technology. Their findings indicate that developers seem willing to adapt the technology to meet client needs, whether by adding requested features or modifying the tool to accommodate the patients’ medical limitations. However, they also seem bound by what is technically feasible. As one developer explained: “Decisions around hardware are enormous...what you can do is gonna be limited to your hardware choices” [96].

As it stands, these constraints disallow developers from meeting all the needs of patients and clinicians. This possibly discourages them from developing a tool at all.

### Domain 4: The Adopter System

This domain entails patient and clinician characteristics that are nonclinical in nature. Because the developer papers did not dive into this, their perspective is not outlined below.

#### Patients

Besides their condition (Domain 1), there are 2 main patient characteristics that influence their willingness to adopt an XR tool.

The most mentioned one is their experience with technology [55-60,62-64,66,67,69,70,72-74,89,112], which can have both positive and negative impacts. Generally, patients who are more experienced with (non-)XR technology have more confidence in their ability to use the tool, find the experience more enjoyable, and need less time to understand it. However, previous technology experience also increases the patient’s expectations of the tool, particularly if they have played highly advanced video games. A negative experience in the past can also demotivate patients from trying another tool again. The second most mentioned patient characteristic is age, mostly because younger patients have more tech experience [56-58,69,74,79,89].

#### Clinicians

While the previous section did not indicate that patient adoption is influenced by clinician characteristics, the opposite is true for clinicians. Rather, 40.5% (15/37) of the clinician papers mentioned how patient characteristics influence the clinician’s decision to adopt.

The 2 main patient characteristics mentioned by clinicians were also the ones mentioned by patients themselves: tech experience and age. As previously indicated, patients with more tech experience have more confidence in using the tool, find the experience more enjoyable, and need less time to get used to it [10,56,84,92,94-96,98,102], and as younger patients generally have more tech experience, they are also more likely to adopt [11,12,56,58,81,84,91,94-96,98,102,110]. As mentioned earlier, clinicians require a minimum number of patients before they can retrieve the financial and time investments associated with adopting XR tools. As a result, clinicians who treat younger or more tech-experienced patients may be more inclined to adopt XR-rehabilitation tools.

A total of 51.4% (19/37) of the papers also mentioned clinician characteristics that impact their likelihood to adopt. Of these characteristics, only experience with technology and age were repeatedly mentioned. As for patients, younger clinicians generally have more tech experience, meaning they better understand the tool’s benefits and need less time to start working with them [10,11,13,56,58,66,79,91,92,96,99-103,107-109]. Characteristics like work experience, education type, and care specialization were also mentioned [10,11,13,100,103,108-110]. However, because these factors were sparsely mentioned, their true impact on adoption remains unclear.

### Domain 5: The Organization

This domain entails how the adoption or development decision is influenced by the health care organization’s capacity and readiness to adopt, the board’s support, and the extent to which established work routines will be disrupted by the XR tools.

#### Patients

None of the patient papers illustrated how the formal health care organization influences the patient’s adoption decision. Hence, it remains unclear whether and how this influences their adoption decision.

However, 15 [55,56,58,64,66,68,71,72,75,77,80,84,87-89] papers did stress the importance of informal caregivers, such as friends and family. The role of these caregivers is twofold. First, they can help patients perform their exercises when they are not able to fully do so themselves, such as by resolving bugs or putting on the HMD. Second, they can motivate patients to try the tool and continue using it by being supportive or even training with them. As an example of how caregivers can *demotivate* patients, one paper [89] illustrated that a participant felt discouraged when her daughter said, “Mom, what are you going to do with a smartphone?”

#### Clinicians

A total of 67.6% (25/37) of the papers illustrated how the health organization impacts the clinician’s adoption decision. The results of these papers can be divided into three categories: (1) resource/time sufficiency, (2) supportiveness, and (3) institute type.

The first 2 characteristics are supported by several sources. First, 22 [11,13,56,58,66,68,73,87,91-93,96,98-100,102,103,105,107-110] papers mentioned that clinicians working in an institute where they get dedicated time and resources are more likely to adopt. As illustrated in the value proposition domain, using XR tools requires both time and money. Naturally, clinicians are unable to adopt the tools if they have insufficient access to either of the 2. Second, 18 [12,13,56,58,87,91-93,96,98-100,102,103,105,107-109] papers illustrated that clinicians working in a supportive environment are more likely to adopt. This has both an administrative component in the form of board support (ie, more resources or time) and a cultural component in which colleagues, lead users, and the board stimulate the clinician to be innovative.

The third characteristic is the institute type. Although it was only mentioned in 4 [107-110] papers, combining it with findings in other domains does indicate that institute type supports XR-adoption in 2 ways. The first way is through private funding. One paper [110] found that clinicians working in private practices have significantly more access to XR tools than those working in public practices. The authors do not explain why this relationship exists. However, as we will illustrate in the following subchapter (the broader domain), public institutions are more dependent on insurance coverage and public funding. Because both funds are sparse, it is likely that private practices have more access to XR tools. The second way is by operating on a sufficiently large scale. Contrary to the aforementioned findings, 2 papers found that most of the XR users work in (public) hospitals and rehabilitation centers [107,109]. These papers also do not explain why this is the case. But, as explained in the clinician’s condition domain, it is plausibly caused by the fact that these institutes operate on a larger scale, meaning they can divide their costs over more patients. This proposition is also supported by 1 [110] paper that found that clinicians working in institutes with more than 100 children per week have significantly more access to XR tools than those working in institutes with less than 100 children per week.

#### Developers

Although the developer papers did not extensively examine the role of the health care organization, 2 studies still gave an indication of their value to developers. One [106] paper indicated that developers could benefit from the endorsement of key health care organizations. Recommendations for safety and success seem important in the market for XR-rehabilitation. Hence, the endorsement of reputable institutes could stimulate other organizations to also use their tools. Another paper [94] indicated that developers seem to not always fully understand the needs of patients and clinicians a priori. By collaborating with health care organizations in the development process, developers can get *“feedback [they] can work with”* [94]. By using this feedback, developers can develop a product with a better value proposition, which plausibly results in more sales.

### Domain 6: The Broader Domain

This domain entails the wider institutional and sociocultural context, such as health policy, insurance reimbursement, and regulatory aspects of patient-facing technological development.

#### Patients

Only 12.5% (5/40) of the papers touched upon the broader domain. A total of 3 of those mentioned that a good internet connection is a necessity for at-home rehabilitation [66,84,88]. This indicates that people living in more technologically advanced regions are better able to use XR-rehabilitation tools. The other 2 [64,89] papers mentioned that some patients are not happy with the societal transition to complex technology. This requires them to continually invest time and money to be able to use new tools, while they are often still satisfied with their older forms of technology.

#### Clinicians

Unlike the patients, there are quite a few clinician papers that touch upon their perspective on the broader domain (18/37 papers, 48.6%). These papers illustrate 3 ways in which the broader domain impacts the clinician’s adoption decision.

The first factor is again the technological advancement of the clinician’s region [31,56,66,84,91,96,97,99,102,104,107,108,110]. The more advanced a region is, the more value an XR tool can provide, for example, because it has a more stable Wi-Fi connection. Clinicians living in more technologically advanced regions are also more exposed to innovations and technology. This means they generally have more tech experience and more opportunities to start using XR-rehabilitation. Mensah-Gourmel et al [110] support this proposition by illustrating that clinicians in Western Europe generally have more access to XR-rehabilitation than clinicians in Northern, Eastern, and Southern Europe.

The second factor is insurance coverage [10,11,91,95,97]. Because new XR hardware can be expensive, clinicians are more likely to adopt a tool if it is reimbursed by the health care insurer. They also want to be covered against insurance claims if injuries were to result from XR-interventions.

The third factor is funding opportunities. Because XR-tools often require a large user base to become financially viable, they currently lack a sustainable revenue model for many clinicians. As a result, their adoption decision heavily depends on the availability of external (public) funding. When such funding is limited or unavailable, clinicians are unlikely to adopt the tools [11,13,92].

#### Developers

Only 1 [106] paper provided insights into the developer’s perspective on the broader domain. Nevertheless, its focus on institutional barriers within the health care industry still yielded some preliminary insights.

First, developers seem to face major barriers in terms of both costs and revenue. When developers develop a tool, they must adhere to strict data management regulations. This potentially contributes to the high development costs mentioned earlier in the developer’s value proposition domain. Even when developers overcome this hurdle, other medical regulations seem to prevent them from scaling up the use of at-home care, because clinicians must evaluate the patient’s condition in the clinic. Additionally, the authors indicate that public clinics “*have no chance of using new solutions*” because health care insurers are hesitant to pay compensation for novel treatment procedures [106]. This arguably narrows the pool of potential customers to private practices, making it less attractive to enter the market.

However, there are also 2 ways by which the broader domain could aid developers. The first one is through collaborations that provide (1) financial support via public funding or (2) recommendations from clinics, informal caregivers, and opinion leaders. The second possible source of support is through the entrance of Big Tech firms. Currently, the market is mostly occupied by start-ups. While typically having less scale and resources than Big Tech firms, these smaller firms are not necessarily opposed to the market entry of these major companies. Rather, they expect that their reputation could change the attitudes of users and could establish a new business ecosystem for current developers. Whether these expectations are true remains to be determined.

### Domain 7: Embedding and Adaptation Over Time

This domain entails how the aforementioned domains evolve over time. Although literature is limited, it still offers some preliminary insights at both the micro and macro levels. At the micro level, the most prevalent insight is that patient adherence tends to decline as sessions progress, but that major differences exist between individuals. Ploderer et al [72] excellently illustrate this by distinguishing three equally sized groups: (1) “failing” patients who stop early, (2) “temporary” patients whose usage fluctuates, and (3) “continuous” patients whose use remains relatively stable over time. Four other papers touch upon this topic by indicating that adherence either decreases [60,84,88] or stays consistent over time [75]. There are indications of what differentiates the 3 use groups, such as the patient’s time and resource availability, but the evidence is currently too sparse to draw robust conclusions. Other micro-level changes are that session efficiency (actual time spent using XR-game/duration of XR-therapy session) increases over time [60] and that clinicians seem to get more confident with XR if they use it more frequently [103]. On the macro level, some actors have indicated what they expect might happen in the future. For example, clinicians expect that they must look for alternatives to address the growing demand for rehabilitation [91], and developers expect that XR’s value as an alternative will continue to grow [106]. Although insightful, it is important to note that these macro-level insights are mostly based on expectations about the future rather than directly observed changes over time.

## Discussion

### Principal Findings

By reviewing the 7 NASSS domains for patients, clinicians, and developers, we provided a first insight into the factors that drive them to use or develop XR-rehabilitation tools. A comparison of the actors shows that their drivers are frequently aligned. For example, all 3 actors embrace tools that motivate patients to exercise more or that allow for home-based therapy with the support of relatives. However, the comparison also reveals 3 instances in which their drivers may not be fully aligned. Because these misalignments represent potential barriers that are underexplored in the current unilateral view on adoption, we discuss them in more detail below. A summary of the main drivers and the resulting misalignments can be found in Multimedia Appendix 3.

### Medical Efficacy

The potential match in drivers between patients and developers seems promising. Some patients do not value medical benefits as much as they value other factors, such as fun. Some patients indicated a preference for full-body exercises over seated exercises, for example, despite the risk of falling or tripping over a cord. Patients also mentioned the importance of a medical evidence base only once and rarely mentioned the risks of overcompensation and addiction.

These drivers are likely to appeal to developers, who are more than able to develop engaging and motivational games. The low need for an evidence base reduces their costs and increases their profit potential. If patients truly attach little value to the medical benefits or evidence, this would be like buying a commercially available exergame like Beat Saber and Gorilla Tag [113]. Hence, a match between the 2 is plausible.

The clinician may not accept this trade-off, however, as it offers insufficient medical benefits. In their work, clinicians are bound by strict medical regulations that prevent them from prescribing tools that do not meet the necessary medical standards [106]. Beyond these regulatory requirements, clinicians also seem to have their own high standards. Overall, 83.8% (31/37) of the clinician papers mentioned that clinicians value the medical efficacy and risks of a tool. A total of 48.6% (18/37) of the clinician papers mentioned that they see the medical evidence base as an essential feature driving their adoption.

The importance of medical efficacy is intuitive, as it is the clinician’s professional and ethical responsibility to provide the best care possible [114]. Therefore, we do not want to put this driver in a negative spotlight. But it does seem plausible that it drives clinicians to *not* prescribe a tool to patients who would have wanted to use it. This even goes for tools that provide medical benefit, but that do not meet the clinician’s medical efficacy standards. Currently, there is no XR-specific research that supports this proposition. But there are indications that it happens for other medical tools, even if they have been approved by medical authorities [115,116].

### The Clinician’s Work Experience

As indicated in the value proposition domain, one benefit of XR-rehabilitation is that it offers autonomy to patients. Instead of having to visit the clinic, they can exercise at a time and location that suits them best. Developers can offer them these tools. It can even be argued that at-home tools are more attractive for developers because economies of scale are easier to achieve when all patients use them at home rather than share one in the clinic.

Various clinicians indicated that they also see enhanced patient autonomy as a benefit, meaning that their interests align with those of patients and developers in this case. However, there is also a set of clinicians for whom treating patients remotely makes their work less enjoyable. They can feel like a virtual coach or a patient mill, rather than a clinician. Using XR can also narrow their skillset to a niche. These losses can be quite substantial. But they are only incurred by clinicians, and not by patients and developers. Because of this, it is theoretically possible that clinicians may decide not to adopt XR, partially because doing so would impede their work experience.

However, in practice this is unlikely to happen as clinicians are highly bound by regulatory frameworks and ethical principles [117]. A more likely consequence is that clinicians will quit their jobs if they experience too much work displeasure [118]. This will not only prevent the patient from using XR but will also further increase staffing problems. Because of this, the impact of XR usage on the clinician’s work experience should be an important consideration for both practitioners and researchers.

### Limited Ability or Willingness to Pay Hinders the Developer’s Economies of Scale

One paper illustrated that developers face “costs of development [that] are still pretty high at present*”* [94]. One sustainable way to recover these costs is to charge the patients and/or clinicians for the tool. However, these payments are a major adoption barrier for both actors. Naturally, patients prefer to pay as little as possible for the tool. Interestingly, clinicians share this perspective because high prices can increase the digital divide among patients [96]. However, clinicians are not always able or willing to carry the full financial burden by themselves. As a result, developers have fewer opportunities to reach sufficient economies of scale to recover their development costs. This plausibly prevents potential developers from entering the market or might cause existing developers to go out of business.

### Theoretical and Practical Contributions

While previous reviews have been valuable in outlining what drives individual actors to not adopt XR-rehabilitation tools, none of them have investigated how their drivers align or misalign. By taking a more holistic point of view and combining these drivers, our findings suggest that a multi-actor view on adoption can provide additional insight into why XR-rehabilitation tools remain sparsely adopted.

We believe that this perspective may apply to other contexts as well. Adoption is also low for other rehabilitation tools, like the Wii, and for XR tools in other medical fields like palliative and mental health care [119-121]. Naturally, the specific reasons for (non-)adoption will vary across settings. For instance, autonomy benefits do not apply to an in-clinic setting, and using a personal phone requires no hardware acquisition. But the core dynamic remains consistent: tools can only be adopted on a large scale if all 3 actors opt in to it. As a result, we believe that a multi-actor perspective is valuable beyond the scope of this paper as well.

Additionally, both governments and entrepreneurs can leverage the concepts and findings in this paper to help reduce pressure on the health care system. For instance, they could consider developing new revenue models. Currently, most developers charge clinicians a 1-time purchase fee or a yearly subscription fee. These revenue models only allow clinicians with a sufficiently large user base to adopt XR tools. But there are other revenue models, such as performance-based risk-sharing arrangements [122] and managed entry agreements [123] that would allow clinicians to only pay for the tool when they use and/or benefit from it. This would also allow small-scale clinics to adopt XR tools. To date, the use of these models has been limited by barriers related to administration, evidence generation, and a lack of transparency [123]. Nonetheless, initiatives like Germany’s new reimbursement process for digital health tools continue to show a positive impact on the adoption of these technologies [124]. Governments could also extend existing orphan drug legislation to relevant digital interventions [125,126]. Our paper also highlights ways in which XR developers can meet the needs of both patients and clinicians. But developers could also innovate beyond that by developing a (sharing) platform, like Netflix and Uber [127]. Such a centralized platform would increase game variety, reduce search time, and reduce per-patient hardware costs by enabling device sharing. To ensure that the development process of these innovations addresses the needs of all 3 actors, we strongly recommend adopting a collaborative approach. Cocreation practices seem especially suitable in this multi-actor context [22,128], and many methods are available. We therefore encourage stakeholders to consult the Health CASCADE database of collaborative methods to determine which one best suits their needs [129].

### Limitations

Although XR-rehabilitation is a novel therapeutic approach, it has been studied across various contexts. To balance the richness and comparability of the included papers, our search query included a wide range of rehabilitation-, device-, and adoption-related search terms, but was also stringent by having a search block that captures behavioral drivers (see the Methods section for further details). Within this methodological trade-off, 2 limitations warrant consideration.

The first limitation is that it resulted in a set of papers with highly diverse research specifications in terms of the disorders treated, device types, intervention types, and usage location (see Multimedia Appendix 2). For example, Wray and Emery [100] conducted a survey where clinicians used an Oculus Quest 2 for drug or alcohol counseling, while Ploderer et al [72] conducted a mixed-methods study in patients with diabetes using their phone to track their foot ulcers at home. As most included studies were single-actor studies (n=49), the misalignments that we identified may potentially stem from these research differences, rather than true discrepancies between actors. At the same time, the low number of studies including all actors (n=1) also further corroborates the importance of future studies taking a multi-actor perspective.

The second limitation concerns the small number of developer papers. Because our review only included 4 such papers [22,94,96,106], the developer findings should be regarded as exploratory rather than conclusive. Although the overall body of developer-focused literature appears limited, the inclusion of the driver-related search terms may have contributed to the low number of included papers.

On top of these 2 query-related limitations, a third limitation is that only 31.3% (20/64) of the included papers used a theoretical framework to investigate adoption behavior. Of those 20 papers, no more than 7 (35%) used the same one (Multimedia Appendix 2). Although using an inductive research approach is valuable for providing new theoretical insights [130], the limited systematic use of concepts and definitions impedes the comparability across papers, thereby making it more difficult to unify them into a coherent conceptual overview.

A fourth limitation concerns the validation of the screening process. As illustrated in the results section, MS, LD, and JK validated the screening process for 219 of the 654 of the initially screened records (33.5%). In this validation sample, they identified 2 false exclusions and 1 false inclusion in the final set of included papers. An extrapolation of this to the nonvalidated set of papers implies that 4 papers may have been falsely excluded and 2 papers may have been falsely included. Although the screening process was conducted nearly perfectly in both the title/abstract stage (κ=0.83) and the full paper stage (κ=0.89), it is important to keep this potential limitation in mind.

The last limitation is that most papers were stringent in their respondent selection criteria by, for example, only selecting patients who had relatively little impairment. Because these people are more likely to adopt XR tools, this paper’s findings might be overly optimistic about the gains of XR, thus minimizing potential losses.

### Future Research Directions

As illustrated in the Results section, the current literature on the adoption of XR-rehabilitation tools contains various opportunities for future research. After reviewing these opportunities, we distilled them into 2 broad directions.

#### Investigating the Developer

As illustrated earlier, very little is known about the developer’s role in the diffusion of XR-rehabilitation tools. To overcome this issue, we suggest that future studies focus on why, when, and how developers can(not) profitably develop and sell XR tools. Because health-tech developers face a wide array of stakeholders [131], we suggest that these papers take a holistic approach and do not solely focus on the interplay with patients and clinicians. Because questions of market entry and profitability also occur beyond health care, we encourage scholars to apply relevant frameworks from other research fields like strategic management. Some exemplary options are the 5 forces model [132], the Political, Economic, Social, Technological, Environmental, and Legal (PESTEL) framework [133], or the resource-based view [134]. Due to the currently limited insights, we also suggest initially using qualitative research methods to get a first understanding of these phenomena.

#### Investigating the 3 Actors Collectively

This work provides a first insight into the conflicts between patients, clinicians, and developers. However, these conflicts remain purely theoretical. Hence, we suggest that future scholars further investigate these 3 actors collectively. These works should focus on empirically validating the 3 conflicts in this paper, discovering other potential conflicts, and finding ways to overcome them.

There are numerous options to validate the 3 conflicts mentioned in this paper. Scholars could take a broad approach, where they determine whether patients and clinicians indeed have different evaluations of the medical benefits, fun, and the clinician’s work experience. But they could also focus on specific features like price and immersion level. Due to the current technological constraints, scholars should also determine which value propositions and features developers are willing and able to develop. Otherwise, the demand cannot be fulfilled by the supply. Considering the current prevalence of qualitative research (see Multimedia Appendix 2), we recommend using more quantitative and experimental research methods for validation research [135,136].

Beyond validating the identified conflicts, future research should also explore other possible conflicts. For example, the developer’s profit goals might incentivize them to make their games as engaging (and addictive) as possible [137]. Some patients might prefer this because they can have more fun. However, clinicians might not prescribe it to patients because they see risks associated with overtraining or misusing the therapy for sole enjoyment purposes. Conflicts may also arise when researchers incorporate additional stakeholders in the analysis. For example, insurance reimbursement is a key adoption driver for clinicians because it removes their payments for hardware and software. But a health care insurer’s desire for efficiency [138] may also pressure clinicians to have fewer physical meetings with patients, which hampers their work experience. Similarly, the high regulatory need to demonstrate safety and medical efficacy when requesting market authorization [139] likely demotivates developers from launching a product. We suggest using qualitative and collaborative methods to get an initial understanding of these theoretically possible but as of yet unexplored misalignments [128,129]. Given the limited insight into how adoption dynamics evolve over time (see Results; Domain 7: Embedding and Adaptation Over Time), we also encourage future research to use longitudinal research designs.

### Conclusions

This review is the first to indicate that the limited adoption of XR-rehabilitation tools can likely not be fully explained by investigating patients, clinicians, and developers in isolation. Rather, we show that bringing these 3 actors together leads to a set of potential misalignments that help explain the low adoption level of these tools. Scholars can use this review as a basis to further investigate the conflicts between these 3 actors or to further investigate the developers’ drivers to invest in XR-rehabilitation tools. Practitioners can also use these findings to create new revenue models, value propositions, and reimbursement regulations.

## Supplementary material

10.2196/80055 Multimedia Appendix 1 Full search query.

10.2196/80055 Multimedia Appendix 2 Descriptives of all included papers.

10.2196/80055 Multimedia Appendix 3 A summary of the main drivers and the resulting misalignments.

10.2196/80055 Checklist 1 PRISMA-ScR checklist.

## Abbreviations

AR: augmented reality
HMD: head-mounted device
MR: mixed reality
NASSS framework: Nonadoption, Abandonment, and challenges to the Scale-up, Spread, and Sustainability framework
PESTEL framework: Political, Economic, Social, Technological, Environmental, and Legal framework
PRISMA-ScR: Preferred Reporting Items for Systematic Reviews and Meta-Analyses for Scoping Reviews
VR: virtual reality
XR: extended reality
