# Supplementary material for: Barriers and Facilitators to the 3 Sides of Extended Reality-Rehabilitation Adoption: Scoping Review
Source: J Med Internet Res. 2026 May 20;28:e80055. doi: 10.2196/80055 (PMC13195264; doi:10.2196/80055)
Supplement: Multimedia Appendix 1 [file jmir-v28-e80055-s001.docx]

# **Multimedia Appendix 1: Full search query**

| **Search engine** | **Search Query** | **Number of hits on 20/01/2026** |
| --- | --- | --- |
| Web of Science (Topic) | **(**Rehab* OR Occupational Therapy OR Physical Therapy OR Speech Therapy OR Language Therapy) AND ("Virtual Reality" OR "Augmented Reality" OR "Mixed Reality" OR "Extended Reality" OR "Head Mounted*" OR Exergam*) AND (Accept* OR Satisf* OR Adopt* OR Uptake OR Utiliz* OR Intention To Try OR Appropriat* OR Perceived Fit OR Relevan* OR Compatib* OR Suitab* OR Useful* OR Usab* OR "Ease of use" OR Practicabil* OR Cost OR Feasib* OR Actual Fit OR Utility OR Fidelity OR Delivered As Intended OR Adher* OR Integrity OR Quality Of Program Delivery OR Implement* OR Penetrat* OR Level Of Institutionalization OR Spread OR Service Access OR Sustainab* OR Maintenance OR Continuation OR Durab* OR Incorpora* OR Integrat* OR Institutionaliz* OR Sustained Usage OR Sustained Use OR Routinization OR Perception* OR Experience* OR Attitude*) AND (Facilitator* OR Barrier* OR Enabler* OR Determinant* OR Driver* OR Hindrance* OR Impediment*) | 350 |
| PubMed | (Rehab*[tiab] OR Rehabilitation [MeSH] OR "Occupational Therapy"[tiab] OR "Occupational Therapy"[MeSH] OR "Physical Therapy"[tiab] OR "Physical Therapy Modalities"[MeSH] OR "Speech Therapy"[tiab] OR "Speech Therapy"[MeSH] OR "Language Therapy"[tiab] OR "Language Therapy"[MeSH]) AND ("Virtual Reality"[tiab] OR "Virtual Reality"[MeSH] OR "Augmented Reality"[tiab] OR "Mixed Reality"[tiab] OR "Extended Reality"[tiab] OR "Head Mounted*"[tiab] OR Exergam*[tiab]) AND (Accept*[tiab] OR "Patient Acceptance of Health Care"[MeSH] OR Satisf*[tiab] OR Adopt*[tiab] OR "Technology Adoption"[MeSH] OR Uptake[tiab] OR Utiliz*[tiab] OR "Intention To Try"[tiab] OR Appropriat*[tiab] OR "Perceived Fit"[tiab] OR Relevan*[tiab] OR Compatib*[tiab] OR Suitab*[tiab] OR Useful*[tiab] OR Usab*[tiab] OR "Ease of use"[tiab] OR Practicabil*[tiab] OR Cost[tiab] OR Feasib*[tiab] OR "Feasibility Studies"[MeSH] OR "Actual Fit"[tiab] OR Utility[tiab] OR Fidelity[tiab] OR "Delivered As Intended"[tiab] OR Adher*[tiab] OR Integrity[tiab] OR "Quality Of Program Delivery"[tiab] OR Implement*[tiab] OR Penetrat*[tiab] OR "Level Of Institutionalization"[tiab] OR Spread[tiab] OR "Service Access"[tiab] OR Sustainab*[tiab] OR Maintenance[tiab] OR Continuation[tiab] OR Durab*[tiab] OR Incorpora*[tiab] OR Integrat*[tiab] OR Institutionaliz*[tiab] OR "Sustained Usage"[tiab] OR "Sustained Use"[tiab] OR Routinization[tiab] OR Perception*[tiab] OR Experience*[tiab] OR Attitude*[tiab]) AND (Facilitator*[tiab] OR Barrier*[tiab] OR Enabler*[tiab] OR Determinant*[tiab] OR Driver*[tiab] OR Hindrance*[tiab] OR Impediment*[tiab]) | 261 |
| Embase | ((rehab*:ab,ti OR 'occupational therapy':ab,ti OR 'physical therapy':ab,ti OR 'speech therapy':ab,ti OR 'language therapy':ab,ti) AND ('virtual reality':ab,ti OR 'augmented reality':ab,ti OR 'mixed reality':ab,ti OR 'extended reality':ab,ti OR 'head mounted*':ab,ti OR Exergam*:ab,ti) AND (accept*:ab,ti OR satisf*:ab,ti OR adopt*:ab,ti OR uptake:ab,ti OR utiliz*:ab,ti OR 'intention to try':ab,ti OR appropriat*:ab,ti OR 'perceived fit':ab,ti OR relevan*:ab,ti OR compatib*:ab,ti OR suitab*:ab,ti OR useful*:ab,ti OR Usab*:ab,ti OR 'Ease of use':ab,ti OR practicabil*:ab,ti OR cost:ab,ti OR feasib*:ab,ti OR 'actual fit':ab,ti OR utility:ab,ti OR fidelity:ab,ti OR 'delivered as intended':ab,ti OR adher*:ab,ti OR integrity:ab,ti OR 'quality of program delivery':ab,ti OR implement*:ab,ti OR penetrat*:ab,ti OR 'level of institutionalization':ab,ti OR spread:ab,ti OR 'service access':ab,ti OR sustainab*:ab,ti OR maintenance:ab,ti OR continuation:ab,ti OR durab*:ab,ti OR incorpora*:ab,ti OR integrat*:ab,ti OR institutionaliz*:ab,ti OR 'sustained usage':ab,ti OR 'sustained use':ab,ti OR routinization:ab,ti OR Perception*:ab,ti OR Experience*:ab,ti OR Attitude*:ab,ti) AND (facilitator*:ab,ti OR barrier*:ab,ti OR enabler*:ab,ti OR determinant*:ab,ti OR driver*:ab,ti OR hindrance*:ab,ti OR impediment*:ab,ti) AND [embase]/lim) | 145 |
| Scopus (Title, Abstract, Keywords) | (Rehab* OR "Occupational Therapy" OR "Physical Therapy" OR "Speech Therapy" OR "Language Therapy") AND ("Virtual Reality" OR "Augmented Reality" OR "Mixed Reality" OR "Extended Reality" OR "Head Mounted*" OR Exergam*) AND (Accept* OR Satisf* OR Adopt* OR Uptake OR Utiliz* OR "Intention To Try" OR Appropriat* OR "Perceived Fit" OR Relevan* OR Compatib* OR Suitab* OR Useful* OR Usab* OR "Ease of use" OR Practicabil* OR Cost OR Feasib* OR "Actual Fit" OR Utility OR Fidelity OR "Delivered As Intended" OR Adher* OR Integrity OR "Quality Of Program Delivery" OR Implement* OR Penetrat* OR "Level Of Institutionalization" OR Spread OR "Service Access" OR Sustainab* OR Maintenance OR Continuation OR Durab* OR Incorpora* OR Integrat* OR Institutionaliz* OR "Sustained Usage" OR "Sustained Use" OR “Routinization” OR Perception* OR Experience* OR Attitude*) AND (Facilitator* OR Barrier* OR Enabler* OR Determinant* OR Driver* OR Hindrance* OR Impediment*) | 408 |

Table 1: Overview of the final search query per search engine

| **Date** | **Initial search or extensions** |
| --- | --- |
| 31/10/2024 | (Rehab* OR Occupational Therapy OR Physical Therapy) AND ("Virtual Reality" OR "Augmented Reality" OR "Mixed Reality" OR "Extended Reality") AND (Accept* OR Satisf* OR Adopt* OR Uptake OR Utiliz* OR Intention To Try OR Appropriat* OR Perceived Fit OR Relevan* OR Compatab* OR Suitab* OR Useful* OR Practicabil* OR Cost OR Feasib* OR Actual Fit OR Utility OR Fidelity OR Delivered As Intended OR Adher* OR Integrity OR Quality Of Program Delivery OR Implement* OR Penetrat* OR Level Of Institutionalization OR Spread OR Service Access OR Sustainab* OR Maintenance OR Continuation OR Durab* OR Incorpora* OR Integrat* OR Institutionaliz* OR Sustained Usage OR Sustained Use OR Routinization) AND (Facilitator OR Barrier OR Enabler OR Determinant OR Driver) |
| 24/10/2025 | *Extension 1:* We added Speech Therapy and Language Therapy to the rehabilitation terms *Extension 2:* We added “Head Mounted*” to the XR terms *Extension 3:* We added Hindrance* and Impediment* to the driver terms *Extension 4:* We added an asterisk at the end of all driver terms *Extension 5:* We have added MeSH terms to the rehabilitation, XR and adoption terms |
| 20/01/2026 | *Extension 1:* We resolved a minor spelling mistake in the adoption terms by changing Compatab* to Compatib* *Extension 2:* We added Exergam* to the XR terms *Extension 3:* We added Usab*, “Ease of Use”, Perception*, Experience* and Attitude* to the adoption terms |

Table 2: Overview of search query extensions during the search period
