# Supplementary material for: Barriers and Facilitators to the 3 Sides of Extended Reality-Rehabilitation Adoption: Scoping Review
Source: J Med Internet Res. 2026 May 20;28:e80055. doi: 10.2196/80055 (PMC13195264; doi:10.2196/80055)
Supplement: Multimedia Appendix 2 [file jmir-v28-e80055-s002.docx]

# **Multimedia Appendix 2: Descriptives of all included papers**

| **Authors** | **Year** | **Actors Treated** | **Qualitative / Quantitative** | **Used Adoption Framework** | **In-Clinic /**  **At-Home** | **Intervention Type** | **Patient Group** | **XR Type** | **Devices** | **Funding source** | **Summary of main findings** |
| --- | --- | --- | --- | --- | --- | --- | --- | --- | --- | --- | --- |
| Slatman et al. [10] | 2026 | Clinicians | Qualitative | None | At-Home | Various | Chronic Lower Back Pain | VR | HMD | ZonMw (case number: 10270032021502) | The authors found four themes related to the role and implementation of VR in clinical practice: (1) Therapeutic VR is perceived as non-threatening to the PT's role, (2) VR has distinctive qualities that complement the physiotherapists toolbox, (3) Contrasting attitudes towards the added value of therapeutic VR, and 4) physiotherapists need specific skills, positive attitudes and knowledge to administer therapeutic VR. |
| MacIntyre et al. [82] | 2026 | Patients Clinicians | Both | CEQ, PEQ and TAQ | In-Clinic | Exercises | Knee Osteoarthritis | VR | HMD | (1) University of South Australia Post Graduate Award, (2) National Health & Medical Research Council Project Grant to TRS (ID1161634), (3) Henry Brodaty Dementia Australia Research Foundation Mid-career Fellowship, (4) book royalties and speaker fees for lectures relating to pain and rehabilitation and (5) a grant from The Hospital Research Foundation (2022–2023) | In study 1, the authors made several changes based on the feedback of patients and clinicians. For example, they added a bike seat option, refined resistance levels and created a step to improve safety when mounting/dismounting the bike. In study 2, the authors found that patients found that patients that biked using the VR-HMD reported higher levels of enjoyment and exercise engagement than those that biked without the VR-HMD. |
| Saidi et al. [22] | 2026 | Patients  Clinicians  Developers | Qualitative | None | In-Clinic | Exercises | Stroke | VR | HMD | No external or research-specific funding | By conducting a thematic analysis of three workshops and ten interviews with patients, clinicians and developers, the authors determined five themes about the participants’ needs and key challenges: (1) adaptability to accommodate stroke-related impairments in home rehabilitation, (2) safety and ease of use as fundamental in VR stroke rehabilitation, (3) goal orientation, (4) motivation, and (5) VR as a complementary tool. |
| Nicora et al. [105] | 2025 | Clinicians | Quantitative | TAM and SUS | Both | Various | Various | Various | Various | Fit4MedRob grant, funded by the Italian Ministry of Research (#PNC0000007) | The authors provided an insight into the clinician’s perspective on VR-based tools and how these perspectives differ between adopters and non-adopters. For example, adopters feel they are more supported by both their colleagues and their supervisors. Using the SUS-questions, the authors also determined that clinicians find VR safe to use and that they would like to frequently use it. |
| Kenea et al. [85] | 2025 | Patients Clinicians | Quantitative | TAM | In-Clinic | Exercises | Stroke | VR | HMD | NASCERE project, funded by VLIR-UOS, Jimma University and Hasselt University | The authors engaged in a collaborative design process with patients, developers, clinicians and other relevant stakeholders to develop a tool that can be used in Low- and Middle-Income Countries. After developing the tool, both patients and clinicians indicated a high perceived ease of use (4.2/5; 4.5/5), high perceived usefulness (4.3/5; 4.7/5) and high acceptance (4.3/5; 4.6/5) of the tool. |
| Pearce et al. [103] | 2025 | Clinicians | Qualitative | None | Both | Various | Various | Various | Various | (1) University of Sydney, (2) Australian National Health and Medical Research Council, (3) The University of Sydney Faculty Research Fund and (4) an unspecified public-private organization which provides rehabilitation services | Respondents indicated that *“clinician uptake of technology did not happen by chance or by the devices simply being made available to clinicians”* (p. 4). Rather, there were three overarching themes that influenced uptake: (1) the investment of time and energy from clinicians, (2) contextual factors and (3) shared understanding and priorities |
| De Vries et al. [60] | 2025 | Patients | Both | None | In-Clinic | Exercises | Various | VR | HMD | No external or research-specific funding | Patients reported high satisfaction with the XR-therapy and moderate exertion levels. Continued use had mixed effects, as factors like session duration increased, while adherence decreased. Reasons for non-adherence to XR-therapy were tiredness, inability to sit up properly, no motivation, no perceived added value or technical difficulties. |
| Stam et al. [61] | 2025 | Patients | Both | USEQ | In-Clinic | Exercises | Various Neurological Conditions | VR | HMD | No external or research-specific funding | Respondents reported a high level of satisfaction, with the median total USEQ score being 27.5. The thematic analysis also indicated that patients enjoyed the game and believed it had various therapeutic benefits. There were also some (minor) barriers, such as a high set-up time, suboptimal ergonomics and technical failures. |
| Felsberg et al. [12] | 2025 | Clinicians | Quantitative | ADOPT-VR2 | Both | Various | Various | Various | Various | No external or research-specific funding | The main finding was that *“most PT and PTA respondents reported they currently do not use VR in clinical practice, but a critical mass are open to exploring its use in the future”* (p. 11). Attitudes, perceived ease of use, compatibility, client-influence, superior influence, perceived behavioural control and self-efficacy were found to play a significant role in the clinicians’ utilization of XR. |
| Mills & Duffy [92] | 2025 | Clinicians | Both | None | Unspecified | Exercises | Autism | VR | Various | Funding source not disclosed in article | Analyzing the (open-ended) survey responses 53 Speech- and Language Therapists resulted in three themes. Firstly, they had a mixed general knowledge of VR, which seemed to negatively influence adoption likelihood. Secondly, they saw both (medical) benefits and risks associated with using VR. Thirdly, they had several support needs, such as trainings and a good evidence base. |
| Robbemond et al. [62] | 2025 | Patients | Qualitative | None | At-Home | Relaxation | Psychiatric Disorders | VR | HMD | The lifestyle medicine grant (project number 50-55515-98-011) and the efficiency research grant (project number 80-85200-98-21015), both from ZonMW. | Based on four focus group sessions, the authors discovered seven themes related to the barriers and facilitators of using a VR-based relaxation tool: (1) perceived usefulness, (2) ease of use, (3) enhancing autonomy, (4) immersive factors, (5) shortcoming initial guidance, (6) insufficient transition back to reality and (7) physical hindrances. |
| Felnhofer et al. [11] | 2025 | Clinicians | Quantitative | None | Unspecified | Exercises | Various | VR | HMD | The Paris Lodron University of Salzburg Publication Fund. | The authors identified four main barrier themes: (1) professional factors, (2) financial factors, (3) therapeutic factors and (4) technological factors. |
| Luc et al. [55] | 2025 | Patients | Qualitative | None | At-Home | Exercises | Neck pain | VR | HMD | The FRIA grant provided by the French Community of Belgium (no. 40014844) | The authors identified that respondents, had mixed perceptions about being at home, had a positive perception of XR, found the tool comfortable and user-friendly and felt immersed/present. They also compared it to conventional exercises and listed several barriers/facilitators to use such as difficulty of completing exercises and freedom to use games. |
| Rathinam et al. [56] | 2025 | Patients  Clinicians | Qualitative | None | In-Clinic | Exercises | Acquired Brain Injury | VR | Various | Topol digital fellowship programme, UK (2022–2023) | The authors identified five major themes related to XR use: (1) training, (2) knowledge, (3) promotion, (4) consideration of barriers such as logistics and (5) family factors. |
| Moeinzadeh et al. [57] | 2025 | Patients | Qualitative | None | At-Home | Exercises | MS | VR | HMD | No external or research-specific funding | The authors found three themes related to the use of XR-exergaming. Firstly, they identified benefits and barriers of physical activity/exercises for fun, fitness and wellness (e.g., engagement and self-efficacy respectively). Secondly, they perceived physical/cognitive improvements. Thirdly, they outlined their experiences with XR, which were both positive (fun and motivation) and negative experiences (e.g., cost and comfort). |
| Miegel et al. [63] | 2025 | Patients | Quantitative | None | In-Clinic | Exercises | OCD  Anxiety | VR | HMD | Forschungsförderungsfonds der Medizinischen Fakultät– Nachwuchsförderung 2022 of the University Medical Center Hamburg-Eppendorf | Respondents were positive about XR, where those using it for public speaking anxiety exposure were more positive than those using it for physical exercise interventions. Some reasons for the positivity were that VR allowed them to demonstrate their skills and that it enhanced their self-efficacy. Some reasons for negativity were distress, hygiene and a limited game variety. |
| Hardeman et al. [64] | 2025 | Patients | Qualitative | Theoretical Framework of Acceptability | At-Home | Exercises | Parkinson’s | AR | HMD | Funding source not disclosed in article | Overall, the patients largely accepted the use of the at-home Augmented Reality tool. The authors also found that (1) considerable variation in perceived effectiveness and effort across individuals, (2) that patients perceived AR-telerehabilitation as an extension, not a replacement, of supervised in-clinic therapy and (3) that adherence in the long-term can be facilitated by facilitating aspects like flexibility and game variation. |
| McDaniel et al. [65] | 2025 | Patients | Both | None | In-Clinic | Relaxation | Unspecified | VR | HMD | The Stanford Chariot Program and SHC Creative and Healing Arts Program. | The 40 respondents were enthusiastic about the tool. 85% would be very likely to recommend the program to another patient and 97.5% indicated that participating in the service had an impact on their time at the hospital. The four main benefits resulting from the open-ended responses were that the tool (1) offered a sense of escape and distraction, (2) had therapeutic benefits, (3) provided an enjoyable experience and that (4) patients enjoyed the interaction with the facilitating staff members. Improvements points were that (1) patients wanted more access/time with the tool and (2) the tool could have more features. |
| Jehl et al. [58] | 2025 | Patients  Clinicians | Qualitative | None | At-Home | Exercises | Chronic pain | VR | HMD | The National Institute of Arthritis and Musculoskeletal and Skin Diseases at the National Institutes of Health (R21AR079140 and K24AR078945-01A1) | The authors listed six main barriers: (1) intervention-identity inconsistency, (2) system-level constraints, (3) lack of guidance from clinicians, (4) research burnout, (5) expectation violation and (6) missing the optimal treatment window. They listed five main facilitators: (1) viewing XR as a bridge to achieving treatment goals, (2) resource accessibility (3) immersion (4) identity-intervention alignment, and (5) champion-level collaborations. |
| Dupont et al. [59] | 2025 | Patients | Qualitative | None | In-Clinic | Relaxation | Complex Mood Disorders | VR | HMD | (1) the Canada First Research Excellence Fund (2) a gift from the Douglas Foundation, (3) a James McGill Professorship and (4) Canadian Institutes of Health Research (Grant 171198, 2020). | Patients were very excited about using a HMD-based tool for relaxation and communication purposes due to the perceived benefits. They also noted some improvement points, such as risks of symptom exacerbation, comfort and time to learn using the tool. |
| Schreiter et al. [91] | 2025 | Clinicians | Qualitative | Diffusion of innovation theory | Both | Exercises | Unspecified | VR | Unspecified | Funding source not disclosed in article | Using an extensive QCA analysis, the authors outlined how adoption drivers are (dis)similar for experienced ‘innovators’ and non-experienced ‘laggards’ of Virtual Reality for rehabilitation purposes. They classified these (dis)similarities based on (1) prior conditions, (2) knowledge state and (3) persuasion stage. One example is that non-experienced clinicians, unlike experienced ones, see patient age as a barrier to adoption. |
| Ramos et al. [66] | 2025 | Patients  Clinicians | Qualitative | None | Unspecified | Exercises | Various | VR | Various | The Ministry of Economy and Innovation (GQ133218) | The respondents noted that XR improves access to physical activities and rehabilitation and that it could allow regular practice of specific movement skills in an engaging manner. They also noted several facilitators (e.g., user-friendliness) and barriers (e.g., lack of resources). |
| Álvarez-Aguado et al. [86] | 2025 | Patients | Qualitative | None | Unspecified | Exercises | Intellectual Disabilities | Various | HMD | The ANID FONDECYT INICIACIÓN (grant 11230525) and FONDEF under (grant ID23I10034). | Respondents indicated both facilitators (e.g., safe training environment and customization) and barriers to adoption (e.g., discomfort and technical complexity). |
| Hewko et al. [67] | 2025 | Patients | Both | COM-B, SSQ, GUESSS and UES | In-Clinic | Exercises | Depression | VR | HMD | (1) The National Research Council Canada’s Canadian Digital Technology Supercluster support program (CDTS-103), (2) the Michael Smith Foundation for Health Research and (3) the National Research Council Canada. | The tools were well-tolerated by participants. They did also not feel the passage of time, compared it to original arcade games and their engagement led to perspiration and heartbeat levels. Respondents had varying preferences for their favourite games. |
| King et al. [68] | 2025 | Patients  Clinicians | Qualitative | None | In-Clinic | Exercises | Musculoskel-etal Pain | VR | HMD | The Junior Co-operative Society of Cincinnati Children’s Hospital Medical Center | The patient’s experiences with XR were categorized under three themes: process of change (e.g., in the form of reduced pain focus), efficacy (e.g., in the form of confidence) and engagement (e.g., in the form of rewards). All therapists agreed that using XR contributed to the patient’s functional improvements. |
| Kühne Escolà et al. [90] | 2024 | Patients  Clinicians | Quantitative | None | In-Clinic | Exercises | Various | VR | HMD | The Projekt DEAL and the Open Access Publication Fund of the University of Duisburg-Essen. | The authors listed several barriers, the most important ones being neuropsychological limitations impeding patients from participating and physical impairments/comorbidities/level of consciousness alterations. Clinicians considered the treatment feasible and motivating but do require supervision. |
| Mensah-Gourmel et al. [110] | 2024 | Clinicians | Quantitative | None | Both | Various | Various | Various | Various | European Academy of Childhood Disability | Years of experience, type of healthcare facility and children’s ages were significant determinants of access to XR. Size of healthcare facility, and ease of access were statistically significant determinants of use of XR. The professional’s age, European subregion were significant determinants of *both* access and use of XR. The authors also listed several barriers, with lack of resources and training were the two main ones. |
| Haghedooren et al. [31] | 2024 | Patients  Clinicians | Both | None | In-Clinic | Exercises | Various | VR | HMD | Interreg NWE/VR4REHAB (EU) and UZ KU Leuven (BE) | Most respondents faced little to no technological issues or discomfort. The tool was generally perceived as easy to use and a useful training device, but there were mixed results on the motivational effect. |
| Groenveld et al. [69] | 2024 | Patients | Both | None | In-Clinic | Exercises | Chest Trauma | VR | HMD | No external or research-specific funding | The respondents indicated that the XR visualization helps to perform exercises and that it immerses them in a different world. The main barriers/facilitators were ease-of-use, patient independence, the hospital environment and integration with usual care. |
| Alrashidi et al. [107] | 2024 | Clinicians | Quantitative | ADOPT-VR2 | Unspecified | Various | Various | Various | Various | Taibah University and Ministry of Education of Saudi Arabia. | Only 7% of the respondents used XR in practice. All VR-users rated the ADOPT-VR2 constructs and the facilitators higher than the non-XR users. Non-experienced respondents rated all barriers higher than experienced respondents. Attitudes toward XR, the compatibility and the peer influence constructs significantly impacted the behavioural intention to use XR. |
| Wu et al. [70] | 2024 | Patients | Qualitative | None | In-Clinic | Exercises | Breast Cancer | VR | HMD | Kaohsiung Chang Gung Memorial Hospital, part of the Chang Gung Medical Foundation, Taiwan (grant CMRPG8L0081) and the China Medical University Project (grant CMU108-MF-21). | The authors identified three main themes: (1) XR was powerful in facilitating rehabilitation, (2) early and repetitive upper limb movements were an advantage of XR rehabilitation and (3) extensive XR use must overcome challenges related to safety, cost and motion sickness. |
| Linge et al. [83] | 2024 | Patients | Qualitative | None | In-Clinic | Exercises | Musculoskeletal diseases Mental health symptoms | VR | HMD | South-Eastern Norway Regional Health Authority | Individuals on long-term sick leave used Beat Saber as part of their occupational rehabilitation. Most participants found it a valuable experience with several benefits, such as it being an enjoyable experience that distracts them from their pain. |
| Roy et al. [93] | 2024 | Clinicians | Quantitative | None | Both | Various | Paediatric | VR/AR | Various | TRAUMA Grant from the CHU Sainte-Justine Foundation | Ease of use was a significant predictor of more favourable usage success, and lack of training and inadequate infrastructure were significant predictors of a less favourable evaluation of usage success. |
| Shiner et al. [13] | 2024 | Clinicians | Quantitative | None | In-Clinic | Various | Various | VR | Unspecified | No external or research-specific funding | Participants indicated support for using XR and had positive views on its utility, potential benefits (beyond routinely available therapies), improvement of the patient’s healthcare experience, patient engagement, and opportunity to learn new skills. They did however feel they lacked support and resources, and they were neutral about having sufficient time to learn about using XR. |
| Polechoński et al. [111] | 2024 | Patients | Quantitative | None | In-Clinic | Exercises | Wheelchair users | VR | HMD | No external or research-specific funding | Both respondents that used XR with and without hand-held weights indicated high satisfaction. The difference between their satisfaction level was insignificant. |
| Twamley et al. [87] | 2024 | Patients  Clinicians | Qualitative | None | In-Clinic | Exercises | Various | VR | HMD | The Lancashire Teaching Hospitals Charity (Charity No. 1051194) and the National Institute for Health Research (NIHR) Applied Research Collaboration North West Coast (ARC NWC). | The authors indicated four main barriers and facilitators to implementing XR. The barriers are: (1) environmental and resource-related, (2) staff competency and confidence, (3) sensory and (4) psychological. The facilitators are: (1) staff training/education, (2) patient assessment/capacity, (3) introduction to technology and (4) intervention support. |
| Cho et al. [108] | 2024 | Clinicians | Quantitative | ADOPT-VR2 | Unspecified | Various | Various | VR | Various | The Technology Innovation Program (Grant Number: 20014480) funded by the Ministry of Trade, Industry, and Energy (MOTIE, Korea) | In this paper, a large number of the respondents (>50%) had experience with using VR/AVG tools. The authors illustrated that the impact of various ADOPT-VR2 constructs (such as social norms, behavioral intention and self-efficacy) significantly differed between clinicians that were (not) experienced with VR/AVG tools. |
| Bradwell et al. [94] | 2023 | Clinicians  Developers | Qualitative | None | In-Clinic | Exercises | Unspecified | VR | HMD, Treadmill & App | (1) UKRI Healthy Ageing Social, Behavioural and Design Research Programme grant number ES/V016113/1, (2) the EPIC (eHealth Productivity and Innovation in Cornwall and the Isle of Scilly) project, part funded by the European Regional Development Fund (3) the University of Plymouth, co-funding EPIC; ERDF Grant number [05R18P02814] and (4) the Innovate grant 10004423 | The authors illustrate the clinician’s perspective on XR in terms of (1) anticipated benefits, (2) acceptability, (3) concerns of use and negative effects, (4) suitability, (5) improvements, and (6) current design. They also illustrate the developer’s perspective on the benefits and challenges of XR as well as their development process. |
| Krishnan et al. [71] | 2023 | Patients | Qualitative | None | At-Home | Various | Stroke | VR/AR | Various | National Institutes of Health Eunice Kennedy Shriver National Institute of Child Health and Human Development (NICHD) R21 HD095138 and National Institute on Disability, Independent Living, and Rehabilitation Research, 90SFGE0002 | The authors found that respondents had limited experience with exergames but were motivated to try if the games were deemed safe, enjoyable and high-fidelity. They also listed a set of facilitators (e.g., presence of feedback and supervision) and barriers (e.g., limited accessibility and training) to adoption. |
| Choukou et al. [88] | 2023 | Patients | Qualitative | None | At-Home | Exercise | Stroke | VR | HMD, Tablet | Gerry McDole in Improved Healthcare Delivery to Rural, Remote, and Underserved Populations of Manitoba | The XR tool was deemed feasible under certain circumstances when used in conjunction with traditional rehabilitation services, despite the presence of some barriers. Some illustrative barriers are anxiety, technical difficulties and the need for training sessions. |
| Brady et al. [95] | 2023 | Clinicians | Qualitative | None | Both | Education  Exercises | Musculosk-eletal Shoulder Pain | VR | HMD | No external or research-specific funding | The respondents’ perspective on XR-rehabilitation was categorized in five themes: (1) immersion in XR reduces fear of movement, (2) enjoyment in XR improves motivation to exercise, (3) certain disorders may benefit more from XR-rehabilitation, (4) there are safety concerns and adverse effects associated with using XR and (5) there are practical constraints for use of XR in clinical practice, such as hygiene, comfort and cost of technology. |
| Brassel et al. [96] | 2023 | Clinicians Developers | Qualitative | None | In-Clinic | Assessment  Exercises | Communic-ation Disorders | VR | HMD | Research Training  Program Scholarship (The University of Sydney and the  Australian Government) and The University of Sydney  Merit Award Scholarship, | The thematic analysis resulted in three themes on the potential of XR in rehabilitation: (1) XR could be a tool that enhances clinical practice, for example by offering a variety of applications in one tool, (2) there is a need to consider the relevant challenges, such as the patient’s physical/cognitive capabilities and (3) there are numerous solutions to pave the way forward, such as a support desk and relevant training. |
| Ploderer et al. [72] | 2023 | Patients | Both | None | At-Home | Tracking self-care | Diabetic foot ulcer | AR | Phone | Queensland University of Technology and the Prince Charles Hospital Foundation, grant number INN2018-36 | Most respondents perceived the tool as valuable, but actual engagement depended on a set of facilitators and barriers. The main facilitators were (1) that the software was installed on the respondent’s personal phone, (2) that respondents were familiar with foot selfies and (3) the presence of dedicated caregivers. The main barriers were (1) work commitments (2) health disruptions (3) lack of confidence, (4) limited accuracy/reliability as well as (5) frustration with lack of healing progress and (6) having to re-do foot checks. |
| Vaezipour et al. [74] | 2023 | Patients | Both | SUS & TAM | In-Clinic | Exercises | Communic-ation Disorders | VR | HMD | Motor Accident Insurance Commission, Australia | Respondents had a positive attitude to the tool, had mixed opinions on its usefulness/ease of use and indicated that intention to use dependent on factors such as training, performance feedback and supervision by a clinician. They also mentioned adoption barriers and facilitators in terms of personal factors, user-friendliness, accessibility and demonstrated benefits. |
| Hosseini et al. [112] | 2023 | Patients | Quantitative | (S)TAM, SOC, UTAUT, FTPS | In-Clinic | Exercises | Long-term care | VR | HMD | No external or research-specific funding | Respondents with gaming experience exhibited higher GS-E scores, but lower PU scores. Due to this, the authors concluded that prior gaming experience influenced XR- acceptance, but that it was not a decisive factor. There was no significant difference between males and females. |
| Cioeta et al. [75] | 2023 | Patients | Qualitative | None | At-Home | Exercises | Chronic pain | VR | HMD & Phone | The Italian Ministry of Health (Ricerca Corrente) | The authors illustrated a small set of facilitators (e.g., being monitored by a clinician) and benefits of XR (e.g., fun, autonomy and gaming with others). |
| Brown et al. [73] | 2023 | Patients  Clinicians | Both | None | In-Clinic | Education  Exercises | Chronic pain | VR | HMD | The Massachusetts General Hospital Research Institute: Bridging Academia with Industry Research Program. | The authors outlined numerous barriers and facilitators to feasibility and adoption of the XR tool. Some exemplary facilitators are patient disorder (e.g., chronicity) and medical benefits. Some exemplary barriers are limited time, medical-side effects and the absence of a support staff. |
| Kulkov et al. [106] | 2023 | Developers | Qualitative | Three pillars of Scott (2013) | Both | Various | Various | VR/AR | Various | Funding source not disclosed in article | The authors listed several facilitators and barriers to the development of XR-rehabilitation tools. Some illustrative facilitators are the entrance of Big Tech (because it increases the market’s legitimacy), recommendations from lead users and the presence of public funding. Some illustrative barriers are the insurer’s reluctance to pay, the government’s data compatibility requirements, and the legal requirement for clinicians to see their patients in-person when assessing their condition. |
| Elor et al. [97] | 2022 | Clinicians | Qualitative | None | At-Home | Various | Various | VR/AR | Various | (1) The National Science  Foundation under Grant No #2037917, (2) the 2020 Seed Fund Award 2020-  0000000044 from CITRIS and the Banatao Institute at the  University of California and (3) the University of California, Santa Cruz Global  Community Health Wellbeings 2020 Fellows Program | Respondents believe that telehealth has benefits and will become an integrated part of physical therapy. But they also see several downsides, such as the lack of hands-on evaluation and limited technological capabilities. They had quite negative opinions about insurance reimbursement, as it is unclear whether it will be granted in the future, it might lower the evidence requirements and turns clinics into “patient mills”. Because of this, several therapists are actively searching for new business models. The respondents also speculated about technology solutions and future directions for telehealth, such as using smartphone-based, wearable sensors and exoskeletons. |
| Tuck et al. [76] | 2022 | Patients | Both | None | In-Clinic | Exercises | Chronic Pain | VR | HMD | Internal Auckland University of Technology research funding | The respondents preferred using XR over the waitlist and regular treatment. Three themes resulted from the interviews: (1) VR is an enjoyable alternative to traditional physiotherapy, (2) VR leads to functional and psychological benefits despite continued pain, (3) and a well-designed VR setup is essential. |
| Chung et al. [98] | 2022 | Clinicians | Qualitative | None | Both | Exercises | OCD | VR | HMD | An Australian Government Research Training Program Scholarship and the David Winston Turner Endowment Fund. | The thematic analysis revealed three broad themes relevant to adoption: (1) organisational factors such as patient engagement, therapeutic efficacy and safety/ethical concerns, (2) organisational factors such as a solid business case, resource challenges and having a service culture, (3) professional factors such as training and staff attitudes. |
| Vaezipour et al. [99] | 2022 | Clinicians | Both | TAM | In-Clinic | Exercises | Communic-ation disorders | VR | HMD | The Motor Accident Insurance Commission (MAIC), Australia | The XR tool had an average usability. Five themes emerged from the thematic analysis: (1) participants had a positive attitude towards the tool, (2) the tool has benefits for both patients and clinicians, (3) the tool’s ease of use is influenced by physical XR characteristics and the simulated environment, (4) respondents would be motivated to use the system within clinical practice, and (5) there were several adoption barriers and enablers, such as training, safety and cost-effectiveness. |
| Cerdán de las Heras et al. [77] | 2022 | Patients | Qualitative | None | In-Clinic | Exercises | COPD | AR | HMD | EUROSTARS and Aarhus University | The authors listed a large amount of relatively raw data, that mostly focused on technological features. Some seemingly important features are comfort, navigation ease and the presence of feedback. |
| Mayer et al. [79] | 2022 | Patients  Clinicians | Qualitative | None | In-Clinic | Exercises | Claustroph-obia  Other anxiety | VR | HMD | (1) The SELFPASS (Self-administered Psycho-Therapy SystemS) project funded by the Federal Ministry of Education and Research (FKZ 13GW0157B), (2) the funding program “Open Access Publikationskosten” of the Deutsche Forschungsgemeinschaft and (3) Heidelberg University | The authors determined eight themes that were relevant for the respondent’s adoption decision and experience: (1) feelings and emotions, (2) personal story, (3) telepresence, (4) positive and negative potential therapeutic effects, (5) barriers such as financial/technical effort, (6) requirements and practical considerations, such as costs and facilities, (7) future prospects, different ways of treatment, and (8) technical realization in terms of the gamification elements, hardware and software. |
| Wray & Emery [100] | 2022 | Clinicians | Quantitative | None | In-Clinic | Exercises | Alcohol / Drug counselling | VR | HMD | The National Institute on Alcohol Abuse and Alcoholism under grant L30AA023336 to TW, and the Brown University School of Public Health under the Catalyst grant program | Overall, respondents indicated that they would be likely to use XR, found it appropriate for their patients, but did rate it lower in terms of feasibility. They also listed a set of barriers, including limited time, having to clean the equipment, limited space and cost. |
| Greenhalgh et al. [101] | 2021 | Clinicians | Both | None | In-Clinic | Exercises | Brain Injury | VR | HMD | (1) The Headache Center for Excellence, (2) the VA polytrauma postdoctoral Fellowship of the VA office of Academic Affairs, (3) Air Force Research Lab (AFRL) | Most participants reported very positive feedback on the XR protocol. They particularly felt that the protocol suited the existing rehabilitation protocols, felt that it had medical benefits, and found it engaging. Usability was determined to be the most enticing piece of the equipment. |
| Reilly et al. [78] | 2021 | Patients | Quantitative | AIM, FIM, SUS | In-Clinic | Exercises | Lower extremity injuries | VR | HMD | Kathryn Cramer Career Development Award #F18KCRAMR | Fifteen patients with operative fractures of the  femur or tibia used a VR-tool to perform heel slide exercises. After performing these exercises, they filled in the AIM, FIM and SUS surveys. Amongst these results came indications that the tool was easy to use, that patients enjoyed the experience and that patients would be open to hybrid forms of physiotherapy. |
| Sánchez-Herrera-Baeza et al. [80] | 2020 | Patients | Both | None | In-Clinic | Exercises | Parkinson’s | VR | HMD | (1) ROBOESPAS (DPI2017-87562-C2-1-R) projects funded by the Spanish Ministry of Economy and Competitiveness-Agencia Estatal de Investigación and (2) the RoboCity2030-III-CM project (S2013/MIT-2748), funded by Programas de Actividades I+D en la Comunidad de Madrid and cofunded by Structural Funds of the EU. | The authors determined four main themes related to the respondent’s experiences. Firstly, respondents felt that the XR treatment did not have more medical benefits than the conventional treatment and saw it as a complement rather than a replacement. The second theme entailed facilitators (e.g., sense of competition and feeling closer to friends/family) and barriers (e.g., fatigue and limited game variety). The third theme outlined how respondents managed and applied the treatment (e.g., getting help from family). The fourth theme entailed potential treatment improvements such as more competitive elements and conducting preparatory sessions to enhance tool familiarity. |
| Aloyuni et al. [104] | 2020 | Clinicians | Quantitative | None | At-Home | Various | Unspecified | VR | Various | No external or research-specific funding | Only 21% of respondents make use of telerehabilitation, and only 3% uses XR for this. Technical issues (24%), staff skill issues (23%), high cost (22%), provider willingness (20%), and location of healthcare institute (10%) were the main barriers to implementation. |
| Tennant et al. [81] | 2020 | Patients  Clinicians | Both | TAM | In-Clinic | Exercises | Cancer | VR | HMD & Phone | The Murdoch Children’s Research Institute Bytes4Health grant and the Royal Children’s Hospital Foundation | The authors inductively determined six main thematic factors that influence XR adoption. These are: (1) perceived benefit to the patient, (2) access to education/training, (3) usability, (4) content, (5) patient safety concerns and (6) equipment safety concerns. |
| Rothgangel et al. [84] | 2019 | Patients Clinicians | Both | TAM | At-Home | Exercises | Phantom Limb Pain | AR | Tablet | The State of North Rhine-Westphalia (NRW, Germany) and the European Union through the NRW Ziel2 Programme as a part of the European Regional Development Fund (Grant No. 005-GW02-035). | This paper outlines the acceptance rates and experiences of both patients and therapists that used a tablet-based AR tool. Overall, the tool was acceptable, but there were some improvement points, such as more reliable technology and more variation and personalization of the exercises and content. |
| Ogourtsova et al. [102] | 2019 | Clinicians | Both | UTAUT | In-Clinic | Assessment & Treatments | Unilateral Spatial Neglect | VR | Unspecified | (1) The Richard & Edith Strauss Fellowship in Rehabilitation Sciences, (2) the Fonds de Recherche du Quebec-Santé (FRQS), (3) the Canadian Institute of Health Research (MOP – 77548) and (4) a Senior Scientist FRQS Salary Award. | The respondents indicated (1) that they had a relatively strong behavioural intention to use XR, (2) that using XR can augment their work goals and patients’ outcomes, (3) that XR could be easy to use or not complicated to use and (4) that they lacked resources and knowledge needed to use XR. |
| Levac et al. [109] | 2017 | Clinicians | Quantitative | ADOPT-VR2 | In-Clinic | Exercises | Various | VR | Various | Funding source not disclosed in article | The most treated patients are those with stroke (25.8%), brain injury (15.3%) and musculoskeletal disorders (14.9%). Therapists with VR/AVG experience rated all ADOPT-VR2 constructs higher than those without experience and the only two constructs that significantly predicted behavioural intention were the tool’s perceived usefulness and the respondent’s self-efficacy. The authors also indicated relevant barriers (e.g., limited space) and facilitators. |
| Seaborn et al. [89] | 2016 | Patients | Qualitative | Needs Analysis Framework | At-Home | Various | Unspecified | MR | Various | The Natural Sciences and Engineering Research Council of Canada | The authors outlined the respondent’s perspective on broad aspects like powered chair use and technology in general, as well as more detailed aspects like game design, interaction style and mixed reality. The main XR-specific finding was that respondents were initially hesitant and saw numerous practical and safety barriers, but that they became interested after further explanation. |
