# Supplementary material for: Barriers and Facilitators to the 3 Sides of Extended Reality-Rehabilitation Adoption: Scoping Review
Source: J Med Internet Res. 2026 May 20;28:e80055. doi: 10.2196/80055 (PMC13195264; doi:10.2196/80055)
Supplement: Multimedia Appendix 3 [file jmir-v28-e80055-s003.docx]

# **Multimedia Appendix 3: A summary of the main drivers and the resulting misalignments**

| Domain | Main patient drivers | Main clinician drivers | Main developer drivers | Potential misalignments found |
| --- | --- | --- | --- | --- |
| The value proposition | Patients value:   1. Their care experience 2. Medical benefits 3. Savings of time/money | Clinicians value the same factors that patients value, but they value them differently. Clinicians also value their work experience, while this is not a relevant factor for patients. | Developers seem to face high development costs | We discovered three potential misalignments:   1. Clinicians have a higher standard for medical efficacy 2. Patients do not value work experience 3. Investing time and money is a loss for patients and clinicians, but developers need an income to retrieve their investments |
| The condition | More impaired patients are less likely to adopt. | More impaired patients are less likely to adopt. Clinicians also need a minimum number of patients that use an XR tool to be able to retrieve their investments. | We found no relevant empirical findings. | Developers can retrieve their investments by charging patients or clinicians. But clinicians are only willing to pay for the tool if they have a minimum number of patients before they are willing to adopt the XR tool. As a result, clinicians treating few patients may not be willing to adopt the tool, even if the patient would like to use it. |
| The technology | There is consensus on a patient’s desire for features like content variety and support staff. There is no consensus on patients’ desire for the level of immersion and the exercise type. | Clinicians largely value the same features as patients. The main difference is that they have a stronger preference for tools with low (medical) risks. | Developers appear willing to develop tools that meet the requirements of patients and clinicians, but they are bound by the current technological possibilities. | Because clinicians prefer tools with low (medical) risks, they may not be willing to prescribe tools that the patients and developers would like to use/develop. Examples include tools for which there is limited medical evidence or tools where patients perform full-body exercises while immersed in Virtual Reality. |
| The adopter system | Patients that are younger and more experienced with technology are generally more likely to adopt XR tools. | Clinicians acknowledge that younger and more tech-experienced patients are more likely to adopt. These same characteristics apply to themselves as well. | We found no relevant empirical findings. | The results did not reveal misalignments between the three actors. |
| The organization | Patients do not mention characteristics of the formal healthcare organization. They do stress the importance of informal caregivers. | The clinician’s decision to adopt is influenced by resource and time sufficiency, supportiveness and institute type. | Developers can greatly benefit from collaborating with healthcare organizations. | The fact that clinicians need sufficient resources and time before they are willing to adopt a tool strengthens the proposition that investing money is a loss for clinicians, while developers need an income to retrieve their investments. |
| The broader domain | We found very few relevant empirical findings. | The clinician’s adoption decision is influenced by the technological advancement of their region, by the insurance coverage and funding opportunities. | Regulatory frameworks appear to mostly hinder developers, but collaborations and the possible entry of Big Tech firms seem to be potential facilitators. | The results did not reveal misalignments between the three actors. |
| Embedding and adaptation over time | Adherence appears to decline over sessions, but major differences exist between patients. | Clinicians gain confidence with XR over time, and they expect a need for alternatives to address the growing demand for care. | Developers expect that XR’s value as an alternative will continue to grow. | The results did not reveal misalignments between the three actors. |
